# Relationship Between Distal and Proximal Neoplasia

**DOI:** 10.1007/s11606-012-2073-x

**Published:** 2012-04-27

**Authors:** Dimitra Dodou, J. C. F. de Winter

**Affiliations:** Department of BioMechanical Engineering, Delft University of Technology, Delft, 2628 CD The Netherlands

*Authors Reply:*--- In a recent meta-analysis, we found that all types of distal lesions are predictive of proximal neoplasia (PN) and that on average 60 % of PN are isolated, that is, not accompanied by distal lesions.^1^ A commentary by Dr. Filik concluded that if all types of distal lesions are predictive of PN, colonoscopy should be performed instead of flexible sigmoidoscopy (FS). We thank Dr. Filik for his comment. In this reply we highlight why FS should not be abandoned in favor of colonoscopy.

Deciding which colon inspection method is most suitable for screening depends on many parameters, including discomfort, complication rates, logistics, and costs.^2^ FS is performed without sedation, leading to more pain than colonoscopy. Colonoscopy has a complication rate that, although low, is 10 times higher than that of FS. Colonoscopy is further accompanied by a loss of three working days for preparing for, undergoing, and recovering from the procedure, compared to half a day in FS.

Colonoscopy has the theoretical advantage over FS that it allows inspection of the proximal colon. Recent population-based studies have reported that, compared to no screening, colonoscopy led to significant reductions of cancer incidence in the distal colon and associated mortality, but its efficacy in the proximal colon was similar to that of FS.^3^ It remains unclear whether these studies failed to show a protective effect of colonoscopy against proximal colon cancer incidence because of poor design or practical limitations (e.g., by including cases in which colonoscopy was performed by non-gastroenterologists), inherent visualization problems of colonoscopy (e.g., miss of flat and pale proximal adenomas), or inherent properties of the proximal colon (e.g., the aggressive nature of proximal tumors). Randomized controlled trials of screening colonoscopy are underway, but their results will not be available before 2021.

In an effort to improve the predictive value of FS, Imperiale et al.^4^ introduced a clinical index including distal findings, gender, and age. The index was applied to a cohort of asymptomatic individuals 50 years or older undergoing screening colonoscopy for the first time and detected 92 % of individuals with advanced proximal neoplasia. Using the index could reduce the number of screening colonoscopies by 40 % as compared to sending everyone to colonoscopy. In our meta-analysis, health characteristics were an important moderator of the association between distal and proximal lesions, with proximal advanced neoplasia being better predicted in asymptomatic populations, young populations, and populations with a low prevalence for proximal advanced neoplasia. Combining the FS outcome with demographics, health characteristics, genetic predisposition, and environmental risks can improve the prediction of PN and strengthen the role of FS as a screening modality. Technological advances in imaging and visualization as well as emerging techniques such as virtual colonoscopy will also contribute to future lesion detection.
